# Discovery of Tricyclic Pyranochromenone as Novel Bruton’s Tyrosine Kinase Inhibitors with In Vivo Antirheumatic Activity

**DOI:** 10.3390/ijms21217919

**Published:** 2020-10-25

**Authors:** Hyewon Cho, Eun Lee, Hye Ah Kwon, Lee Seul, Hui-Jeon Jeon, Ji Hoon Yu, Jae-Ha Ryu, Raok Jeon

**Affiliations:** 1 College of Pharmacy, Sookmyung Women’s University, Cheongpa-ro 47-gil 100, Yongsan-gu, Seoul 04310, Korea; hy-0802@sookmyung.ac.kr (H.C.); eunlee178@gmail.com (E.L.); kwonhea91@gmail.com (H.A.K.); ryuha@sookmyung.ac.kr (J.-H.R.); 2 New Drug Development Center, Daegu-Gyeongbuk Medical Innovation Foundation, 80 Chembok-ro, Dong-gu, Daegu 41061, Korea; autrition15@dgmif.re.kr (L.S.); hjjeon@dgmif.re.kr (H.-J.J.); yujihoon@dgmif.re.kr (J.H.Y.)

**Keywords:** pyranochromenone, BTK inhibitor, irreversible inhibitor, rheumatoid arthritis

## Abstract

Bruton’s tyrosine kinase (BTK) is an attractive target for treating patients with B cell malignancies and autoimmune diseases. Many BTK inhibitors have been identified; however, like other kinase inhibitors, they lack diversity in their core structures. Therefore, it is important to secure a novel scaffold that occupies the adenine-binding site of BTK. We screened an in-house library of natural products and their analogs via a biochemical assay to identify a novel scaffold for targeting BTK. A pyranochromenone scaffold, derived from a natural active component decursin, was found to be effective at targeting BTK and was selected for further optimization. A series of pyranochromenone analogs was synthesized through the modification of pyranochromenone at the C7 position. Pyranochromenone compounds with an electrophilic warhead exhibited promising BTK inhibitory activity, with IC_50_ values in the range of 0.5–0.9 µM. A docking study of the representative compound **8** provided a reasonable explanation for compound activity. Compound **8** demonstrated good selectivity over other associated kinases and decreased the production of proinflammatory cytokines in THP cells. Moreover, compound **8** presented significant in vivo efficacy in a murine model of collagen-induced arthritis.

## 1. Introduction

Bruton’s tyrosine kinase (BTK) belongs to the Tec family of nonreceptor tyrosine kinases, which are predominately expressed in immune cells, including B cells, macrophages, monocytes, basophils, and mast cells, but not in mature plasma cells and T cells [1,2]. BTK plays a critical role in B cell receptor (BCR) signaling in B cells, where it regulates their survival, activation, proliferation, differentiation, and maturation [3,4]. It is also essential for the activation of mast cells and macrophages via FcγR and FcεR, the Fc receptors for IgG and IgE, respectively, which in turn induce the release of proinflammatory mediators [5,6,7,8]. Therefore, BTK is a promising target for novel therapeutic interventions in various immunological disorders, including rheumatoid arthritis, asthma, and systemic lupus erythematosus [9,10,11].

**Figure 1:**
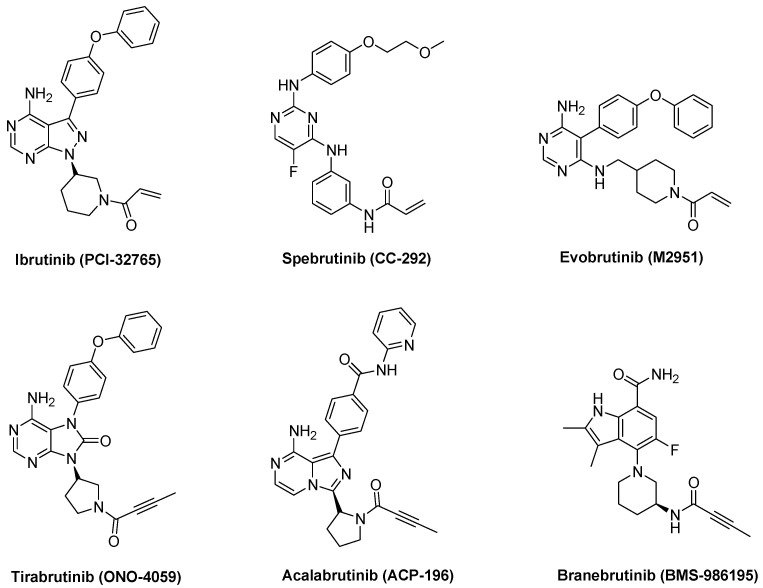
Representative irreversible BTK inhibitors.

Several BTK inhibitors have been developed in the past few decades, which can be classified into two groups, reversible and irreversible. The irreversible inhibitors ibrutinib (PCI-32765) [12], spebrutinib (CC-292, AVL-292) [13], tirabrutinib (GS-4059, ONO-4059) [14], acalabrutinib (ACP-196) [15], and evobrutinib (M2951) have advanced to clinical development (Figure 1). Ibrutinib (PCI-32765, Imbruvica), the first Food and Drug Administration (FDA)-approved covalent irreversible BTK inhibitor, has been used successfully for the treatment of patients with relapsed or refractory mantle-cell lymphoma (MCL) and chronic lymphocytic leukemia (CLL). Second-generation irreversible BTK inhibitors are currently in clinical trials and are being developed for the treatment of patients with autoimmune disorders and B cell malignancies. Irreversible BTK inhibitors covalently bind to Cys481 in the active site of BTK. Cys481 is situated at the conserved kinase site of Tec family kinases (TEC, BMX, ITK, and TXK), EGFR, ERBB2, ERBB4, JAK3, BLK, and MKK7. Therefore, there is a need for the development of selective BTK inhibitors with a favorable safety profile, especially for the treatment of chronic autoimmune disorders.

**Figure 2:**
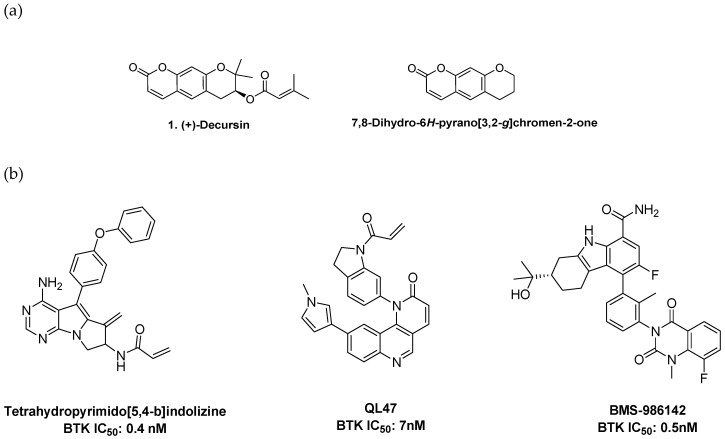
(**a**) Structures of decursin and pyranochromenone. (**b**) Reported tricyclic BTK inhibitors.

Since the ATP-binding site in kinases is highly conserved, many BTK inhibitors share high similarities in their monocyclic or bicyclic hinge-binding cores. To explore novel BTK hinge-binding scaffolds, we screened a focused in-house library consisting of bioactive compounds derived from natural products and their synthetic derivatives. Some of the screened compounds presented BTK inhibitory activity; decursin was selected for further study. Decursin is a major active component of *Angelica gigas* and is well known for its biological activities, including anti-inflammatory, anticancer, antioxidative, and antiangiogenic properties [16,17,18]. Although decursin has many pharmacological roles, target-based medicinal chemistry approaches have not been sufficiently implemented. Decursin was of particular interest since its core structure is a tricyclic pyranochromenone, a unique core structure in the research and development of kinase inhibitors (Figure 2a). A few tricyclic BTK inhibitors have been reported. Recently, two interesting tricyclic BTK inhibitors were introduced: a pyrimidopyrrolizine analog transformed from pyrazolopyrimidine, the bicyclic hinge binder of ibrutinib, by ring merging, and a benzonaphthyridinone analog, in which the nitrogen of naphthyridinone interacts with a hinge residue (Figure 2b). These findings suggest that the selected pyranochromenone scaffold would be a suitable starting point for targeting BTK. Of particular interest, we expected that the cyclic ester moiety of pyranochromenone could act as a hinge binder, which is a unique type of hinge binder not found in other kinase enzymes.

**Figure 3:**
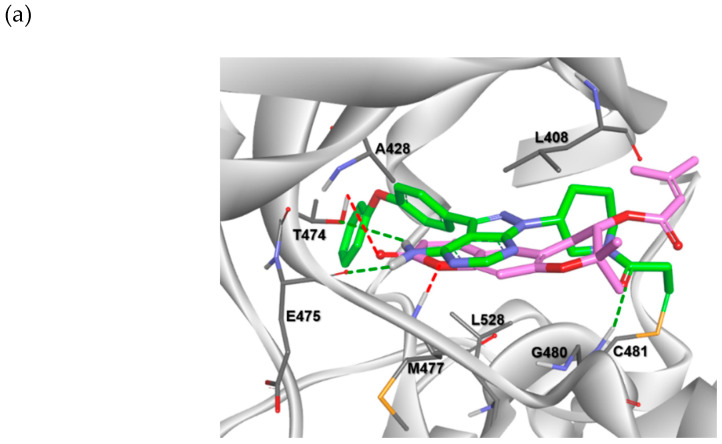

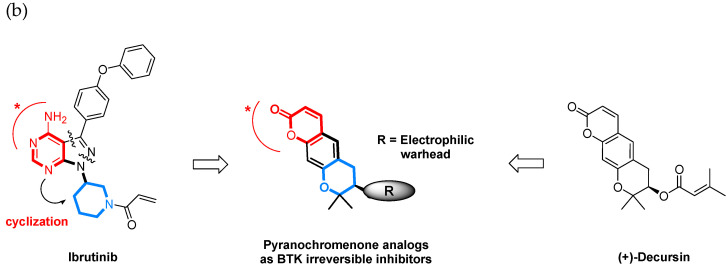
(**a**) Superposition of a docked pose of decursin (magenta) and the crystal structure of ibrutinib (green) in the active site of BTK (PDB code: 5P9J); (**b**) design of novel irreversible pyranochromenone-based BTK inhibitors. * Interacting groups at the hinge region.

First, we investigated whether the pyranochromenone scaffold could be used for the discovery of BTK inhibitors through a docking study. Decursin was docked in the active site of BTK cocrystallized with ibrutinib (Protein Data Bank (PDB) code: 5P9J), and its binding pose was compared with that of ibrutinib (Figure 3a). The results demonstrated that the pyranochromenone core of decursin could be properly situated in the active site of BTK, with a conformation similar to that of ibrutinib. Pyranochromenone has also been shown to interact with gatekeeper Thr474 and Met477 in the binding region. These docking results supported our hypothesis on the role of the dihydropyranone moiety of pyranochromenone as a hinge binder. We speculated that a novel irreversible BTK inhibitor could be developed by introducing a warhead at an appropriate position in pyranochromenone to target the Cys481 residue of BTK (Figure 3b). Herein, we report the design and synthesis of novel irreversible pyranochromenone-based BTK inhibitors with in vivo efficacy in a murine model of rheumatoid arthritis.

## 2. Results and Discussion

### 2.1. Synthesis of Pyranochromenone Analogs

**Scheme 1:**
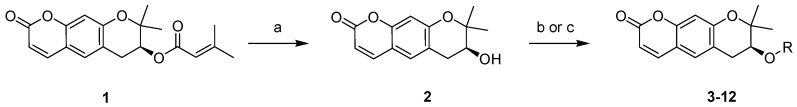
Reagents and conditions: (**a**) KOH, EtOH, 7 h, reflux; (**b**) alkyl or alkoxylcarboxylic acid, DCC, DMAP, DCM, 5–7 h, RT; (**c**) acyl chloride, TEA, MC, 1–2 h, RT.

The target compounds (2–12) were prepared as depicted in Scheme 1. The ester underwent a simple modification, or an electrophilic warhead was introduced at the C7 position of pyranochromenone. Decursin (compound **1**) was isolated from *A. gigas* and hydrolyzed under basic conditions to obtain decursinol (compound **2**). Target ester compounds **3–8** were obtained through a coupling reaction between compound **2** and appropriate acids, using dicyclohexylcarbodiimide (DCC) as a coupling reagent in the presence of 4-dimethylaminopyridine (DMAP). Target compounds **9–12** were generated by treating compound **2** with acyl chloride in the presence of triethylamine.

### 2.2. Structure–Activity Relationship Analysis

The inhibitory potential of pyranochromenone compounds **1–12** against the enzymatic activity of BTK was analyzed employing the HotSpot kinase assay platform, which is similar to that previously reported [19]. The percentage BTK inhibition and IC_50_ values, compared with the positive control, ibrutinib, are summarized in Table 1, and the IC50 curves for compounds **8**, **9** and **10** are provided in Figure S1. Decursin (compound **1**) presented weak inhibitory activity against BTK, whereas decursinol (compound **2**) was inactive. This demonstrates that the presence of an appropriately sized substituent at C7 could enhance the inhibitory activity of the compounds. However, the introduction of the but-2-enoyl (**3**) and 4-bromobut-2-enoyl (**4**) substituents at the C7 alcohol of decursinol conferred minimal inhibitory activity. Furthermore, compounds carrying the cinnamoyl (**5**) and 4-fluorocinnamoyl (**6**) substituents had no BTK inhibitory activity.

To target the Cys481 residue in the active site of BTK, we introduced warhead Michael acceptors; but-2-ynoyl (**7**), acryloyl (**8**), and ethenesulfonyl (**9**) groups; and an alkyl chloride (**10**) at the C7 position of pyranochromenone. As anticipated, compounds **8–10**, which contained the electrophilic warhead substituents, exhibited promising BTK inhibitory activity, with IC_50_ values in the range of 0.5–0.9 µM. However, compound **7**, which contained the but-2-ynoyl substituent, presented relatively low BTK inhibitory activity.

To confirm the role of the electrophilic warhead groups, the acryloyl group of compound **8** was replaced with methylacryloyl (**11**) and propionyl (**12**) groups, which are less electrophilic or lack an electrophilic feature. The BTK inhibitory activity of compounds **11** and **12** was completely diminished. The significant difference in the inhibitory activity of compounds **8** and **12** could explain the critical role of the acryloyl moiety in potentiating the BTK inhibitory activity of compound **8**. These results suggested that compound **8** and other compounds with an introduced warhead could covalently bind the Cys481 residue in the active site of BTK, ensuring potent and irreversible inhibition. It was also confirmed by comparison of the inhibitory activity of the representative compounds (**8**–**10**) with electrophilic warheads against wild-type BTK (BTK-wt) and mutant BTK (BTK-C481S). The tested compounds (**8–10**) lost their inhibitory activity against BTK-C481S, unlike BTK-wt, suggesting that the tested compounds exhibited irreversible activity through covalent bonds to C481.

Many BTK inhibitors have proven highly effective; however, a number of off-target effects have been reported due to the inhibition of other tyrosine kinases, such as EGFR, ITK, TEC, and BMX [20]. Therefore, efforts have focused on developing new compounds with good selectivity profiles. Compound **8**, with an acryloyl warhead, was selected for further biological evaluation since the acryloyl group was the warhead of ibrutinib, which was used as a key comparative compound. We assessed the selectivity of compound **8** over EGFR and TEC family kinases, which possess a conserved cysteine residue in the ATP-binding site. The selectivity profile of compound **8** is presented in Table 2. Compound **8** was selective against BTK. The biochemical activities of compound **8** differed among TEC family kinases. Compound **8** exhibited weak inhibitory activity against ITK and moderate activity against TEC, but strong inhibitory activity against BMX and TXK. The inhibitory potential of compound **8** differed between members of the EGFR family. Compound **8** exhibited weak inhibitory activity against EGFR and was inactive against ERBB4. The inhibitory activity of compound **8** against JAK3 was also low. Overall, compound **8** was found to be selective for BTK over ITK, EGFR, ERBB4, and JAK3.

### 2.3. Molecular Docking Analysis

**Figure 4:**
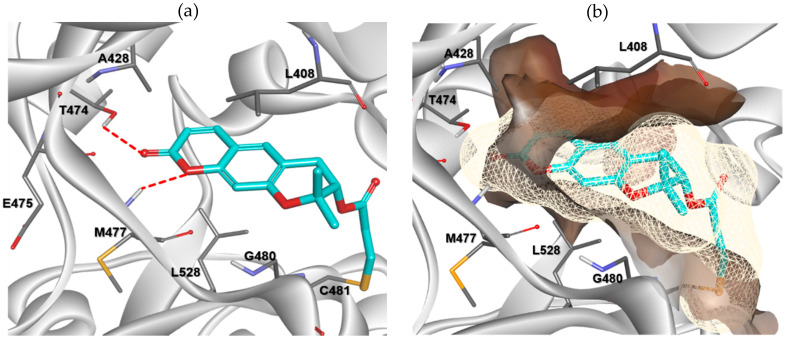
Predicted binding mode of compound **8** (cyan) with BTK (PDB code: 6O8I). (**a**) Key interactions of compound **8** with BTK. H-bonds to the protein are shown, denoted by red dashed lines. The side chains of the active site residues are represented by white sticks. (**b**) The hydrophobicity map surrounding compound **8** in the active site of BTK. The hydrophobic interactions are shown by surface representation.

Docking analysis and visualization were performed using SYBYL-X version 2.1.1 (Tripos inc., St. Louis, UT, USA), Cresset Flare V3 (Cresset inc., Lerchworth, UK)., and Discovery Studio Visualizer Biovia inc., San Diego, CA, USA). The binding pose and interactions of compound **8** with BTK (PDB code: 6O8I) are depicted in Figure 4. Compound **8** was properly situated in the active site of BTK, as expected from the results of previous docking studies. Compound **8** formed two hydrogen bonding interactions with BTK. The oxygen at position 1 of pyranochromenone formed a hydrogen bond with the backbone of Met477 in the hinge region. The oxygen of the carbonyl group formed hydrogen bonds with the gatekeeper residue Thr474 as well as with Met477. In addition, the pyranochromenone core formed hydrophobic interactions with Leu408, Ala428, and Leu528. The tetrahydropyran ring was placed in a narrow hydrophobic H2 pocket formed by Leu408 and Gly480. The gem-dimethyl group on tetrahydropyran fit well into the hydrophobic pocket and interacted with Leu408. Importantly, the acryloyl moiety was covalently bound to the mercapto group of Cys481 in the active site of BTK via a Michael addition reaction. As observed in the Structure-activity relationship (SAR) study, the role of the acryloyl group was verified by confirming that the BTK inhibitory activity was lost in compound **12**, in which a saturated propionyl group was introduced instead of an acryloyl warhead. Collectively, the biological activity data were supported by the docking simulations. 

### 2.4. Permeability and CYP Inhibitory Activity

Owing to its promising biochemical and cellular activity, the in vitro permeability and CYP inhibitory activity of compound **8** were further characterized. The PAMPA assay showed that the permeability of compound **8** was excellent. In a panel of cytochrome P450 isoforms, compound **8** rarely inhibited CYP1A2 and CYP3A4, weakly inhibited CYP2D6, and moderately inhibited CYP2C9 and CYP2C19 (Table 3).

### 2.5. In Vitro Inhibitory Activity on Cytokine Production

**Figure 5:**
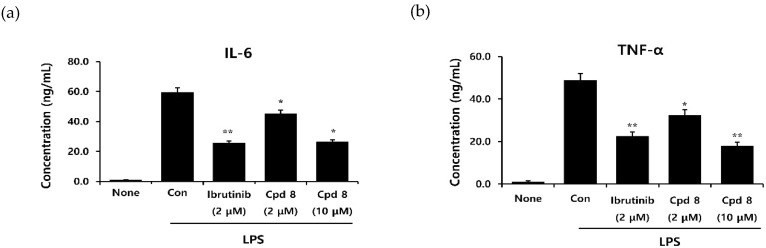
Effects of compound **8** on lipopolysaccharide (LPS)-induced human IL-6 (**a**) and TNF-α (**b**) release in THP-1 cells. Differentiated THP-1 cells were treated with compound **8** and then cotreated with LPS for 24 h. The levels of proinflammatory cytokines were determined using ELISA. * *p* < 0.05, ** *p* < 0.05, and *** *p* < 0.005 indicate significance, according to the *t*-test.

BTK plays a critical role in high-affinity IgE receptor (FcεRI) and B cell receptor (BCR) signaling, resulting in the release of proinflammatory cytokines. Therefore, BTK inhibitor treatment can reduce the production of both lymphoid and myeloid cytokines [21]. A study performed on human pro-monocytic THP-1 cells demonstrated that lipopolysaccharide (LPS)-activated BTK engages the NF-κB pathway, resulting in the production of several proinflammatory cytokines [22]. We examined the cellular effects of compound **8** on the production of cytokines IL-6 and TNF-α in LPS-stimulated THP-1 cells. Following treatment with LPS alone or LPS and compound **8**, the levels of cytokines secreted by THP-1 cells were assessed (Figure 5). LPS increased the output of proinflammatory cytokines. Compound **8** significantly and dose-dependently reduced the production of both TNF-α and IL-6. The inhibitory activity of compound **8** at 10 μM was comparable with that of ibrutinib at 2 μM.

### 2.6. Antirheumatic Activity in a Murine Model of Collagen-Induced Arthritis (CIA)

**Figure 6:**
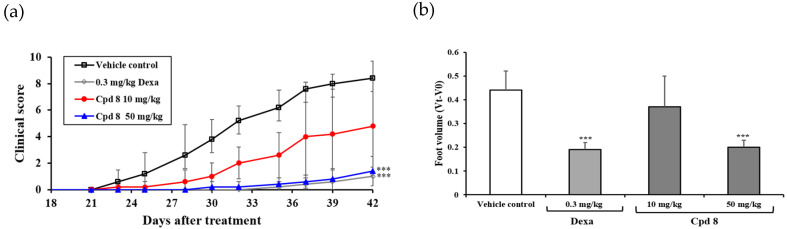
Efficacy of compound **8** in a murine model of collagen-induced arthritis (CIA). (**a**) Mean clinical scores following daily oral treatment with compound **8**, on days 21–25, 28–32, and 35–39, with different doses of compound **8** and dexamethasone are represented as the mean ± SD (*n* = 5). Mice with CIA in the control group received the vehicle only. Clinical scores following treatment with 0.3 mg/kg dexamethasone or 10 and 50 mg/kg of compound **8** differed significantly from those with the control. * *p* < 0.05, ** *p* < 0.01, and *** *p* < 0.001 indicate a significant difference, according to the *t*-test. (**b**) Paw swelling. Refer to the Experimental Section for experimental details.

The in vivo efficacy of compound **8** was evaluated in a murine inflammatory model of collagen-induced arthritis (CIA). The study included four groups: a vehicle-treated control group and three treatment groups. The treatment groups comprised a dexamethasone-treated group, in which dexamethasone served as the positive control, and two treatment groups, in which animals were treated with compound **8** at doses of 10 and 50 mg/kg. Compound **8** was administered once daily for 15 days by oral gavage, and the in vivo efficacy was evaluated by measuring the clinical score and foot volume. As depicted in Figure 6, compound **8** significantly reduced the severity of CIA in a dose-dependent manner. The efficacy of compound **8** at a dose of 50 mg/kg was comparable to that of dexamethasone at 0.3 mg/kg. There were no significant changes in the body weights of animals in all the treatment groups (data not shown) during treatment. Compound **8** markedly improved the severity of arthritis in the murine model of CIA with no obvious toxicity.

## 3. Materials and Methods 

### 3.1. Chemistry

Most reagents and solvents used in this study were purchased from Sigma-Aldrich (St. Louis, MO, USA) chemicals, and used without further purification. Unless indicated, all anhydrous solvents were distilled under nitrogen gas. Ethyl alcohol, dichloroethane, and triethylamine were distilled from calcium hydride under nitrogen. Column chromatography was performed using silica gel 60 (230–400 mesh, Merck, kenilworth, NJ, USA). Thin-layer chromatography (TLC) was performed using Kieselgel 60 F254 plates (Merck, kenilworth, NJ, USA). The infrared (IR) spectra were recorded on a JASCO FT/IR 430 spectrophotometer. The NMR spectra were recorded on a Bruker 500 spectrometer (^1^H 500 MHz and ^13^C 125 MHz). The chemical shifts are expressed in parts per million (ppm, δ) relative to that of the internal standard, tetramethylsilane. The ^1^H-NMR data are represented in the order of the chemical shift, multiplicity (s, singlet; d, doublet; t, triplet; q, quartet; m, multiplet and/or multiple resonances), number of protons, and coupling constant in hertz (Hz).

#### 3.1.1. Preparation of Decursin (1) 

Dried *A. gigas* was purchased from the Corporation (Seoul, Republic of Korea) in 2015. *A. gigas* (300 g) was extracted with 1.9 L of 95% ethanol at 80 °C for 4 h in a two-neck flask. The extract was collected and filtered to remove precipitates. The filtrate was concentrated and redissolved in 500 mL of ethanol at 40 °C for 2 h, following which the mixture was cooled to room temperature to form precipitates. The resulting precipitates were filtered and washed with cold chloroform and ethyl acetate. The residue was purified by column chromatography (*n*-hexane/EtOAc = 2:1) to obtain 5.1 g of decursin (compound 1); yellow oil, ^1^H NMR (500 MHz, CDCl_3_) δ 7.78 (1H, d, *J* = 10. Hz), 7.32 (1H, s), 6.69 (1H, s), 6.18 (1H, d, *J* = 10.0 Hz), 5.61 (1H, s), 5.08 (1H, t, *J* = 5.0 Hz), 3.21 (1H, dd, *J* = 16.8, 4.8 Hz), 2.85 (1H, dd, *J* = 16.8, 4.8 Hz), 2.14 (3H, s), 1.88 (3H, s), 1.38 (3H, s), 1.32 (3H, s).

#### 3.1.2. *S*-(+)-Decursinol (2)

KOH (333.2 mg, 5.94 mmol) was added to a solution of compound **1** (1.3 g, 3.96 mmol) in EtOH (7.9 mL) with stirring at 0 °C and then stirred overnight at 60 °C. The reaction was quenched with ice water, and the solution was adjusted to pH 2 with a 2N HCl solution. The aqueous mixture was then extracted thrice with ethyl acetate (10 mL). The organic layer was dried over anhydrous magnesium sulfate and concentrated in vacuo to obtain the crude product. The residue was then poured into cold chloroform, filtered, and washed with MeOH to afford the pure product 2 as a white solid. Yield: 48.2%, ^1^H NMR (400 MHz, CD_3_OD) δ 7.82 (1H, d, *J* = 9.2 Hz), 7.34 (1H, s), 6.68 (1H, s), 6.19 (1H, d, *J* = 9.2 Hz), 3.81 (1H, dd, *J* = 16.4, 5.6 Hz), 3.09 (1H, dd, *J* = 16.4, 5.6 Hz), 2.79 (1H, dd, *J* = 16.4, 5.6 Hz), 1.36 (3H, s), 1.32 (3H, s); ^13^C NMR (100 MHz, CD_3_OD) δ 163.5, 158.4, 155.3, 145.7, 130.5, 119.3, 114.0, 113.2, 104.8, 79.7, 69.7, 31.5, 25.9, 21.7.

#### 3.1.3. General Procedure for the Synthesis of Compounds 3–7, 11, and 12

A mixture of the appropriate acids (1.0 equiv), DCC (2.0 equiv), and 4-DMAP (1.0 equiv) was dissolved in anhydrous dichloromethane. (S)-(+)-decursinol (compound **2**, 1.0 equiv) was subsequently added, and the reaction mixture was stirred at room temperature for 5–10 h. The progress of the chemical reaction was determined by TLC. When the (S)-(+)-decursinol spot was no longer visible on the TLC plate, the mixture was concentrated in vacuo, and the residue was purified by flash silica gel column chromatography using an ethyl acetate/hexane solvent system as eluent.

(7*S*)-(+)-But-2-enoic acid, 8,8-dimethyl-2-oxo-6,7-dihydro-2*H*,8*H*-pyrano[3,2-g]chromen-7-ylester (3)

Yield: 34.6%, caramel form, ^1^H NMR (500 MHz, CDCl_3_) δ 7.59 (1H, d, *J* = 10.0 Hz), 7.16 (1H, s), 7.02–6.94 (1H, m), 6.79 (1H, s), 6.23 (1H, d, *J* = 10.0 Hz), 5.84 (1H, d, *J* = 15.0 Hz), 5.11 (1H, t, *J* = 5.0 Hz), 3.20 (1H, dd, *J* = 15.0, 5.0 Hz), 2.88 (1H, dd, *J* = 15.0, 5.0 Hz), 1.87 (2H, d, *J* = 5.0 Hz), 1.39 (3H, s), 1.37 (3H, s); ^13^C NMR (125 MHz, CDCl_3_) δ 165.9, 161.2, 156.4, 154.2, 145.9, 143.1, 128.6, 122.1, 115.7, 113.3, 112.8, 104.7, 76.6, 69.8, 27.8, 24.9, 23.1, 18.0.

(7*S*)-(+)-But-2-enoic acid, 8,8-dimethyl-2-oxo-6,7-dihydro-2*H*,8*H*-pyrano[3,2-g]chromen-7-yl ester (4)

Yield: 61.9%, colorless oil, ^1^H NMR (500 MHz, CDCl_3_) δ 7.59 (1H, d, *J* = 10.0 Hz), 7.16 (1H, s), 7.04–6.98 (1H, m), 6.80 (1H, s), 6.23 (1H, d, *J* = 10.0 Hz), 6.04 (1H, d, *J* = 15.0 Hz), 5.13 (1H, t, *J* = 5.0 Hz), 3.99 (2H, d, *J* = 5.0 Hz), 3.22 (1H, dd, *J* = 15.0, 5.0 Hz), 2.90 (1H, dd, *J* = 15.0, 5.0 Hz), 1.39 (3H, s), 1.37 (3H, s); ^13^C NMR (125 MHz, CDCl_3_) δ 164.8, 161.2, 156.2, 154.2, 143.1, 142.9, 128.6, 123.9, 115.4, 113.4, 112.9, 104.8, 76.5, 70.6, 28.8, 27.7, 24.9, 23.2.

(7*S*)-(+)-*trans*-Cinnamic acid, 8,8-dimethyl-2-oxo-6,7-dihydro-2*H*,8*H*-pyrano[3,2-g]chromen-7-yl ester (5)

Yield: 72.9%, white solid, ^1^H NMR (500 MHz, CDCl_3_) δ 7.69 (1H, d, *J* = 16.4 Hz), 7.59 (1H, d, *J* = 9.2 Hz), 7.52–7.49 (2H, m), 7.39–7.36 (3H, m), 7.18 (1H, s), 6.84 (1H, s), 6.43 (1H, d, *J* = 16.4 Hz), 6.24 (1H, d, *J* = 8.4 Hz), 5.21 (1H, t, *J* = 4.8 Hz), 3.26 (1H, dd, *J* = 16.8, 8.4 Hz), 2.95 (1H, dd, *J* = 16.8, 8.4 Hz), 1.43 (3H, s), 1.40 (3H, s); ^13^C NMR (125 MHz, CDCl_3_) δ 166.3, 161.2, 156.3, 154.2, 145.8, 143.1, 133.9, 130.6, 128.9, 128.7, 128.1, 117.3, 115.7, 113.3, 112.9, 104.7, 76.6, 70.2, 27.8, 24.9, 23.3.

(7*S*)-(+)-*trans*-4-Fluorocinnamic acid, 8,8-dimethyl-2-oxo-6,7-dihydro-2*H*,8*H*-pyrano[3,2-g]chromen-7-yl ester (6)

Yield: 59.3%, white solid, ^1^H NMR (500 MHz, CDCl_3_) δ 7.64 (1H, d, *J* = 16.0 Hz), 7.59 (1H, d, *J* = 9.2 Hz), 7.51–7.48 (2H, m), 7.18 (1H, s), 7.09–7.04 (2H, m), 6.83 (1H, s), 6.35 (1H, d, *J* = 16.0 Hz), 6.24 (1H, d, *J* = 9.2 Hz), 5.20 (1H, t, *J* = 4.8 Hz), 3.25 (1H, dd, *J* = 17.2, 4.4 Hz), 2.94 (1H, dd, *J* = 17.2, 4.4 Hz), 1.44 (3H, s), 1.39 (3H, s); ^13^C NMR (125 MHz, CDCl_3_) δ 166.0, 164.9, 162.9, 161.1, 156.4, 154.1, 144.5, 143.1, 130.2, 130.1, 129.9, 128.7, 117.1, 116.2, 115.9, 113.4, 112.9, 104.7, 76.6, 70.2, 27.9, 24.9, 23.3.

(7*S*)-(+)-But-2-yonic acid, 8,8-dimethyl-2-oxo-6,7-dihydro-2*H*,8*H*-pyrano[3,2-g]chromen-7-ylester (7)

Yield: 17.6%, caramel form, ^1^H NMR (500 MHz, CDCl_3_) δ 7.58 (1H, d, *J* = 10.0 Hz), 7.16 (1H, s), 6.80 (1H, s), 6.24 (1H, d, *J* = 10.0 Hz), 5.12 (1H, t, *J* = 5.0 Hz), 3.20 (1H, dd, *J* = 15.0, 5.0 Hz), 2.90 (1H, dd, *J* = 15.0, 5.0 Hz), 1.97 (3H, s), 1.40 (3H, s), 1.37 (3H, s); ^13^C NMR (125 MHz, CDCl_3_) δ 161.2, 156.2, 154.3, 143.1, 128.7, 115.3, 113.5, 112.9, 104.9, 87.1, 76.3, 71.9, 71.6, 27.6, 24.9, 23.0, 3.9.

(7*S*)-(+)-2-Methylacrylic acid, 8,8-dimethyl-2-oxo-6,7-dihydro-2*H*,8*H*-pyrano[3,2-g]chromen-7-yl ester (11)

Yield: 54.1%, caramel form, ^1^H NMR (500 MHz, CDCl_3_) δ 7.58 (1H, d, *J* = 10.0 Hz), 7.16 (1H, s), 6.80 (1H, s), 6.23 (1H, d, *J* = 10.0 Hz), 6.07 (1H, s), 5.58 (1H, s), 5.09 (1H, t, *J* = 5.0 Hz), 3.22 (1H, dd, *J* = 15.0, 5.0 Hz), 2.89 (1H, dd, *J* = 15.0, 5.0 Hz), 1.91 (3H, s), 1.40 (3H, s), 1.38 (3H, s); ^13^C NMR (125 MHz, CDCl_3_) δ 166.5, 161.2, 156.3, 154.2, 143.1, 135.8, 128.6, 126.4, 115.7, 113.3, 112.8, 104.7, 76.6, 70.6, 27.7, 25.0, 22.9, 18.2.

(7*S*)-(+)-Propionic acid, 8,8-dimethyl-2-oxo-6,7-dihydro-2*H*,8*H*-pyrano [3,2-g]chromen-7-ylester (12)

Yield: 31.4%, white solid, ^1^H NMR (500 MHz, CDCl_3_) δ 7.59 (1H, d, *J* = 10.0 Hz), 7.16 (1H, s), 6.79 (1H, s), 6.23 (1H, d, *J* = 10.0 Hz), 5.06 (1H, t, *J* = 5.0 Hz), 3.19 (1H, dd, *J* = 15.0, 5.0 Hz), 2.85 (1H, dd, *J* = 15.0, 5.0 Hz), 2.37–2.31 (1H, m), 1.38 (3H, s), 1.36 (3H, s), 1.13 (1H, t, *J* = 5.0 Hz); ^13^C NMR (125 MHz, CDCl_3_) δ 173.8, 161.2, 156.3, 154.1, 143.1, 128.6, 115.7, 113.3, 112.8, 104.7, 76.5, 69.9, 27.7, 27.6, 24.9, 22.9, 9.0.

#### 3.1.4. General Procedure for the Synthesis of Compounds 8–10

Triethylamine (1.0 equiv) was added to a solution of decursinol 2 (1.0 equiv) in dichloromethane at 0 °C and stirred for 30 min. Then, acyl chlorides (1.5 equiv) were added dropwise. The mixtures were stirred at room temperature for 2–4 h, and the reactions were monitored by TLC (*n*-hexane/EtOAc = 2:1). The reaction was quenched with water and extracted with dichloromethane (10 mL × three), washed with brine, and dried over anhydrous magnesium sulfate. The solvent was removed under reduced pressure, and the residue was purified by column chromatography to obtain compounds 8–10.

(7*S*)-(+)-Acrylic acid, 8,8-dimethyl-2-oxo-6,7-dihydro-2*H*,8*H*-pyrano [3,2-g]chromen-7-yl ester (8)

Yield: 31.2%, caramel form, ^1^H NMR (500 MHz, CDCl_3_) δ 7.59 (1H, d, *J* = 10.0 Hz), 7.16 (1H, s), 6.80 (1H, s), 6.41 (1H, d, *J* = 20.0 Hz), 6.23 (1H, d, *J* = 10.0 Hz), 6.11 (1H, dd, *J* = 20.0, 10.0 Hz), 5.86 (1H, d, *J* = 10.0 Hz), 5.13 (1H, t, *J* = 5.0 Hz), 3.23 (1H, dd, *J* = 15.0, 5.0 Hz), 2.90 (1H, dd, *J* = 15.0, 5.0 Hz), 1.40 (3H, s), 1.38 (3H, s); ^13^C NMR (125 MHz, CDCl_3_) δ 165.8, 161.5, 156.6, 154.5, 143.4, 132.2, 128.9, 128.2, 115.9, 113.7, 113.2, 105.0, 76.8, 70.6, 28.0, 25.2, 23.4.

(7*S*)-(+)-Ethenesulfonic acid, 8,8-dimethyl-2-oxo-6,7-dihydro-2*H*,8*H*-pyrano [3,2-g]chromen-7-yl ester (9)

Yield: 34.1%, white solid, ^1^H NMR (500 MHz, CDCl_3_) δ 7.56 (1H, d, *J* = 10.0 Hz), 7.16 (1H, s), 6.73 (1H, s), 6.58 (1H, dd, *J* = 17.0, 10.0 Hz), 6.42 (1H, d, *J* = 17.0 Hz), 6.20 (1H, d, *J* = 10.0 Hz), 6.14 (1H, d, *J* = 10.0 Hz), 4.71 (1H, t, *J* = 5.0 Hz), 3.25 (1H, dd, *J* = 15.0, 5.0 Hz), 3.12 (1H, dd, *J* = 15.0, 5.0 Hz), 1.39 (3H, s), 1.37 (3H, s); ^13^C NMR (125 MHz, CDCl_3_) δ 161.3, 115.7, 154.3, 142.9, 132.9, 130.3, 128.6, 114.7, 13.7, 113.2, 104.9, 77.9, 76.2, 28.9, 25.1, 22.7.

(7*S*)-(+)-Chloroacetic acid, 8,8-dimethyl-2-oxo-6,7-dihydro-2*H*,8*H*-pyrano [3,2-g]chromen-7-ylester (10)

Yield: 32.7%, caramel form, ^1^H NMR (500 MHz, CDCl_3_) δ 7.59 (1H, d, *J* = 10.0 Hz), 7.16 (1H, s), 6.80 (1H, s), 6.25 (1H, d, *J* = 10.0 Hz), 5.13 (1H, t, *J* = 5.0 Hz), 4.06 (2H, d, *J* = 8.0 Hz), 3.23 (1H, dd, *J* = 15.0, 5.0 Hz), 2.90 (1H, dd, *J* = 15.0, 5.0 Hz), 1.40 (3H, s), 1.37 (3H, s); ^13^C NMR (125 MHz, CDCl_3_) δ 166.8, 161.1, 156.0, 154.2, 143.0, 128.6, 115.0, 113.5, 113.0, 104.8, 76.2, 72.3, 40.8, 27.6, 24.8, 23.1.

### 3.2. In Vitro Kinase Enzymatic Assays

Kinase enzymatic assays for BTK, BMX, TXK, TEC, ITK, EGFR, ERBB2, ERBB4, and JAK3 were performed at Reaction Biology Corporation (www.reactionbiology.com, Malvern, PA, USA) using the HotSpot assay platform. Briefly, specific kinase/substrate pairs and the required cofactors were prepared in a reaction buffer comprising 20 mM HEPES (pH 7.5), 10 mM MgCl_2_, 1 mM EGTA, 0.02% Brij35, 0.02 mg/mL BSA, 0.1 mM Na_3_VO_4_, 2 mM DTT, and 1% DMSO. The compounds were added to the reaction mixture and allowed to incubate. Approximately 20 min later, a mixture of ATP (Sigma-Aldrich, St. Louis, MO, USA) and ^33^P ATP (PerkinElmer, Waltham, MA, USA) was added to the reaction mixture at a final concentration of 10 μM. The reactions were performed at room temperature for 120 min; subsequently, the reactions were spotted onto P81 ion exchange filter papers (Whatman Inc., Maidstone, UK). Any unbound phosphate was removed by thoroughly washing the filter papers with 0.75% phosphoric acid. After subtracting the background data derived from the control reactions with the inactive enzyme, kinase activity was expressed as the percentage kinase activity remaining in the test samples compared with that of the reaction setups composing the vehicle (dimethyl sulfoxide) only. GraphPad Prism software was used to obtain the IC_50_ values and perform curve fitting.

### 3.3. Molecular Modeling

Molecular modeling calculations and visualizations were performed using Surflex-Dock, interfaced with SYBYL-X software version 2.1.1 (Tripos inc., St. Louis, MO, USA) on a system with Windows 7 Home Premium K 64-bit OS and Discovery Studio Visualizer (Biovia inc., San Diego, CA, USA). The flexibility of the ligands, proteins, and biomolecules was considered in this automated docking program. The compounds were constructed in an incremental fashion, where each new fragment was added in all possible positions and conformations to a preplaced base fragment inside the active site. All molecules used for docking were first sketched in SYBYL and subjected to energy minimizations using the Tripos force field and Gasteiger–Huckel charge, with 100,000 iterations of the conjugate gradient method using a convergence criterion of 0.05 kcal/mol. For protein preparation, all the hydrogens and MMFF02 charges were added, and the side chain amides were fixed. A staged minimization was performed using the Tripos force field and MMFF02 charges with 10,000 iterations of the Powell method, with a convergence criterion of 0.5 kcal/mol without initial optimization. Three-dimensional (3D) coordinates of the active sites were determined from the X-ray crystal structure of BTK (PDB code: 6O8I), complexed with branebrutinib. Covalent docking of compound **8** to the active site of BTK was performed using Cresset Flare V3 software (Cresset inc., Cambridgeshire, UK). For docking, the protein was prepared and minimized using Cresset Flare V3 software, and the grid box was defined according to the clustered ligand, branebrutinib, in the downloaded PDB file. The docking calculations were performed in Cresset Flare V3 software in covalent docking mode.

### 3.4. Cytokine Assay

THP-1 cells were obtained from the American Type Culture Collection (ATCC, Manassas, VA, USA). The cells were cultured in RPMI-1640 supplemented with 10% FBS, 1% P/S, and 0.05 mM 2-mercaptoethanol and maintained at 37 °C and 5% CO_2_ in a humidified incubator. Cytokine secretion (hIL-6 and TNF-α) in the culture supernatants of the THP-1 cells was measured using a human cytokine ELISA kit (Abcam). Differentiated THP-1 cells were treated with agents and LPS for 24 h, after which the cell culture media was centrifuged at 2000× *g* for 10 min to remove debris. Briefly, each sample (50 μL) or standard was mixed with the appropriate detection antibody and incubated for 1 h at room temperature on a plate shaker. In addition, 100 μL of TMB development buffer was added to the sandwich complexes and incubated in the dark for 10 min. Subsequently, 100 μL of stop solution was added to each well, and the optical density (OD) was measured at 450 nm. GraphPad Prism 6.07 was used for statistical analyses (San Diego, CA, USA). All *p*-values were derived from unpaired two-tailed *t*-test. Statistical significance was indicated as either *; *p* < 0.05 or **; *p* < 0.005.

### 3.5. In Vitro Permeability Assay

GIT-PAMPA was performed according to the manufacturer’s instructions (Pion Inc., Billerica, MA, USA). Briefly, the test compound was diluted in donor buffer (pH 7.4) to obtain a concentration of 50 μM. Then, 200 μL of this solution was added to the lower part of a 96-well PAMPA sandwich plate. The transmembrane side of the donor part was coated with a GIT-0 lipid solution, and 200 μL of the acceptor buffer was added to the upper part of the PAMPA sandwich plate. After incubating for 4 h at 25 °C, each sample was transferred to a fresh UV plate, after which the UV spectra were measured at wavelengths of 250–498 nm, and the permeability rate (Pe, 10^−6^ cm/sec) was analyzed using the Pion PAMPA Explorer software (version 3.8, Pion Inc., Billerica, MA, USA).

### 3.6. In Vitro CYP Inhibition Assay

The potential for CYP450 inhibition (CYP1A2, 2C9, 2C19, 2D6, and 1A2) was assessed using Promega P450-Glo^TM^ CYP450 assay systems. Luciferin-ME served as the substrate for CYP1A2, and the inhibitor α-naphthoflavone was used as a positive control. Luciferin-H was used as the substrate for CYP2C9, and the inhibitor sulfaphenazole was used as a positive control. Luciferin-H EGE was selected as the substrate for CYP2C19, whereas the inhibitor amitriptyline served as a positive control. Luciferin-ME EGE was used as the substrate for CYP2D6, whereas the inhibitor quinidine served as a positive control. Luciferin-BE was used as the substrate for CYP3A4, whereas the inhibitor ketoconazole was used as a positive control. The assays were performed using the excitation and emission wavelengths recommended by the manufacturer. Briefly, a CYP enzyme and a P450-Glo™ substrate were combined in potassium phosphate (0.1 M KPO_4_) buffer, with or without a test compound of interest, and the reaction was initiated by adding an NADPH regeneration system. The reaction was initiated by adding two volumes of 2 times concentrated NADPH regeneration system. The solutions on the plate were mixed for 10 s on an orbital shaker and incubated at room temperature for 20 min. Luminescence was subsequently measured using a Tecan Infinite M1000 PRO microplate reader (Tecan Group Ltd., Switzerland). The percent inhibition was calculated relative to that of the positive control at a fixed concentration. For more information pertaining to the assay protocol, please refer to the instructions for the kits (P450-Glo™ CYP1A2 Screening System—Cat. No. V9770; P450-Glo™ CYP2C9 Screening System—Cat. No. V9790; P450-Glo™ CYP2C19 Screening System—Cat. No. V9880; P450-Glo™ CYP2D6 Screening System—Cat. No. V9890; and P450-Glo™ CYP3A4 Screening System—Cat. No. V9920 from Promega, USA).

### 3.7. In Vivo Antirheumatic Activity

The animal study was approved by the Institutional Animal Care and Use Committee (IACUC) of the Korea Research Institute of Bioscience and Biotechnology on March 15, 2017, under approval number KRIBB-AEC-17090. Male DBA1/JNCrli mice (7 weeks old) were purchased from Japan CRL, Inc. The mice were housed in specific pathogen-free (SPF) conditions and were allowed ad libitum access to food and water. After 7 days of acclimatization, the mice were immunized with 0.05 mL of a type II collagen emulsion (Hooke Kit^TM^ Chicken Collagen, Cat. No. EK-0210 from Hooke) and Freund’s complete adjuvant by subcutaneous injection at 1.5 cm distal from the base of the tail. After 21 days, the immunized mice were administered a booster injection with 0.05 mL of a type II collagen emulsion (Hooke Kit^TM^ Chicken Collagen, Cat. No. EK-0211 from Hooke) and Freund’s incomplete adjuvant. The emulsions were prepared according to the manufacturer’s instructions. On day 21, the mice with CIA were randomized into four groups. The animals in the four groups were administered either 0.3 mg/kg dexamethasone (*n* = 5), 10 mg/kg compound **8** (*n* = 5), 50 mg/kg compound **8** (*n* = 5), or vehicle (*n* = 5), once a day, for 3 weeks, orally. The vehicle, in which all test compounds were suspended, comprised DMAC (10%), Tween 80 (10%), and DW (80%). Clinical arthritis scores and body weights were assessed three times per week for 15 days. The paw volumes were measured using a LE7500 plethysmometer (Panlab, Barcelona, Spain) on days 0 and 42.

## 4. Conclusions

In summary, a series of BTK inhibitors, based on the pyranochromenone scaffold, were designed and synthesized. The synthesized compounds potentially inhibited the enzymatic activities of BTK, with IC_50_ values ranging between 0.5 and 0.9 μM. The results of a SAR study indicated that the introduction of electrophilic warheads to the pyranochromenone scaffold was critical for the BTK inhibitory activity of the compounds. The representative compound **8** was subjected to further evaluation. The results demonstrated that compound **8** presents good kinase selectivity over Tec and EGFR family kinases, potentially inhibits the production of proinflammatory cytokines, and confers significant in vivo efficacy in a murine model of CIA. Further efforts to improve the potency and selectivity will focus on targeting BTK-specific pockets by introducing hydrophobic tails into the pyranochromenone scaffold. Structural optimizations on this attractive lead candidate are underway.

## Figures and Tables

**Table 1:**
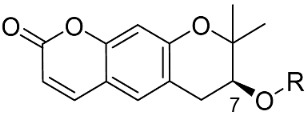
Effect of R substituents on BTK inhibition.

Compound No.	R	% Inhibition (10 µM) ^a^		IC_50_ (µM) ^b^
		BTK-wt	BTK-C481S	
1	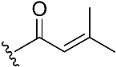	32.0		>10 µM
2	H	7.7		>10 µM
3	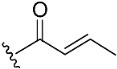	11.3		>10 µM
4	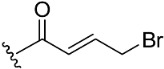	20.3		>10 µM
5	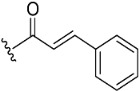	NA ^c^		>10 µM
6	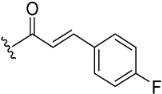	NA		>10 µM
7	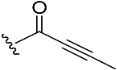	34.5		7.0
8	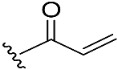	98.2	9.7	0.8
9	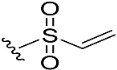	95.8	15.8	0.9
10	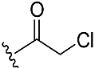	99.2	18.6	0.5
11	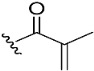	NA		>10 µM
12	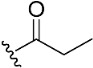	NA		>10 µM
Ibrutinib ^d^	-	99.6		0.00018

^a^ Percentage BTK inhibition was determined and compared with that of the negative control (DMSO) at 10 µM. The values represent the mean values of two separate experiments. ^b^ The IC_50_ values were calculated using GraphPad Prism version 5.0. ^c^ NA indicates not active at 10 µM. ^d^ Ibrutinib as a positive control.

**Table 2:** Kinase selectivity profile of compound **8**.

Kinase	% Inhibition at 10 µM ^a^
BTK	98.2
BMX	95.4
TXK	88.5
TEC	54.5
ITK	17.3
EGFR	32.9
ERBB2	59.2
ERBB4	NA ^b^
JAK3	13.2

^a^ Percentage BTK inhibition compared with that of the negative control (DMSO) at 10 µM. The values represent the mean values of two separate experiments. ^b^ NA indicates not active at 10 µM.

**Table 3:** Permeability and CYP inhibition of compound **8**.

**Permeability ^a^**		−4.06 ± 0.064
CYP inhibition ^b^	1A2	<1
	2C9	66.1
	2C19	48.2
	2D6	18.9
	3A4	<1

^a^ PAMPA assay (Pe, cm/s): high > −4.07; medium −4.07 to −4.87; low < −4.87. ^b^ % inhibition of human CYP isozymes at 10 μM.

## Supplementary Materials

Supplementary materials can be found at https://www.mdpi.com/1422-0067/21/21/7919/s1. Figure S1: The concentration dependent IC50 curves of the compounds 8, 9 and 10.

## Abbreviations

CYP	Cytochrome P450
DMSO	Dimethyl sulfoxide
DMAC	Dimethylacetamide
DW	Distilled water
PAMPA	Parallel artificial membrane permeability assay
LPS	Lipopolysaccharide
