# Supplementary material for: Discovery of Tricyclic Pyranochromenone as Novel Bruton’s Tyrosine Kinase Inhibitors with In Vivo Antirheumatic Activity
Source: Int J Mol Sci. 2020 Oct 25;21(21):7919. doi: 10.3390/ijms21217919 (PMC7663272; doi:10.3390/ijms21217919)
Supplement: Supplementary file 1 [file ijms-21-07919-s001.pdf]

Supplementary Materials

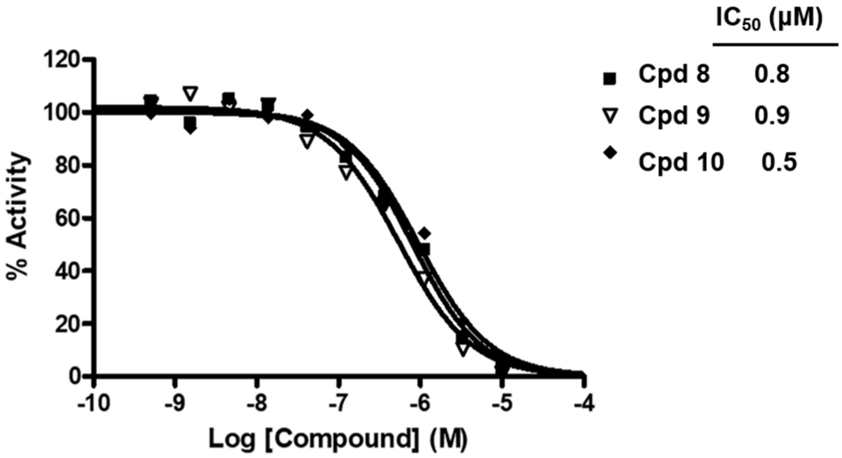

Figure S1. The concentration dependent IC50 curves of the compounds **8**, **9** and **10**
